# Production Is Only Half the Story — First Words in Two East African Languages

**DOI:** 10.3389/fpsyg.2017.01898

**Published:** 2017-10-30

**Authors:** Katherine J. Alcock

**Affiliations:** Department of Psychology, Fylde College, Lancaster University, Lancaster, United Kingdom

**Keywords:** language acquisition, vocabulary acquisition, Bantu languages, East Africa, Communicative Development Inventories

## Abstract

Theories of early learning of nouns in children’s vocabularies divide into those that emphasize input (language and non-linguistic aspects) and those that emphasize child conceptualisation. Most data though come from production alone, assuming that learning a word equals speaking it. Methodological issues can mean production and comprehension data within or across input languages are not comparable. Early vocabulary production and comprehension were examined in children hearing two Eastern Bantu languages whose grammatical features may encourage early verb knowledge. Parents of 208 infants aged 8–20 months were interviewed using Communicative Development Inventories that assess infants’ first spoken and comprehended words. Raw totals, and proportions of chances to know a word, were compared to data from other languages. First spoken words were mainly nouns (75–95% were nouns versus less than 10% verbs) but first comprehended words included more verbs (15% were verbs) than spoken words did. The proportion of children’s spoken words that were verbs increased with vocabulary size, but not the proportion of comprehended words. Significant differences were found between children’s comprehension and production but not between languages. This may be for pragmatic reasons, rather than due to concepts with which children approach language learning, or directly due to the input language.

## Introduction

### What Are Children’s First Spoken Words?

Children first learning to say words in a variety of languages tend to produce names for things (Gentner, 1982; Goldfield and Reznick, 1990; Au et al., 1994; Caselli et al., 1995; Bassano, 2000; Kauschke and Hofmeister, 2002; Bornstein et al., 2004; McDonough et al., 2011). Different schools of thought have put forward a variety of different explanations for this “noun bias.”

Some authors suggest that this is due to children having a set of pre-existing biases including an object bias (Markman, 1990). Others conclude that biased output may be a consequence of input. Influences may include the types of referents, and their correspondences, that are present in child- (and adult-) directed speech (Gleitman et al., 2005). Both schools of thought appear to assume that there are robust and important differences between children’s core knowledge of nouns and of other types of words.

Most data though are obtained from production rather than comprehension, so it is not certain that this is representative of children’s underlying knowledge. In fact, even some classic papers including Goldin-Meadow et al. (1976) and Gentner (1982) suggest that bias toward nouns is possibly weaker in comprehension.

Given these theoretical suggestions, it is important to determine whether nouns and verbs are both represented in early word knowledge. Researchers need to investigate systematically a variety of languages, looking at both early comprehension and early production. It is also important to examine a variety of cultural settings. We cannot answer questions such as these by only carrying out research in Western settings or on European languages.

I will now assess further evidence for a predominance of nouns in spoken words; I will then turn to the first words comprehended. I will address differences and similarities between languages and cultures, as the literature so far has findings of both.

#### Linguistic Variance in First Spoken Words

It is possible that noun bias is language-specific. Choi and Gopnik (1995) suggested that sentence-final verbs in Korean leads to verbs having greater salience. They conclude that Korean-learning language children learn verbs earlier than in other languages, in preference to nouns. Brown (1998) examined the spontaneous speech of children learning Tzeltal, a Mayan language, and concluded that an early appearance of verbs may be due to the richness of meaning carried by many verbs. She observes that at the one word stage children’s utterances mainly consist of single verbs whose meanings are close to those conveyed by nouns in other languages. In addition, the very earliest words were observed, as in many languages, to be words for people.

Tardif and colleagues (Tardif et al., 1997, 1999) found that the proportion of nouns or verbs appearing in children’s early vocabulary in English and Mandarin was dependent on the method used to collect data. They noted that more verbs appeared in spontaneous speech than in parent-completed vocabulary checklists. Tardif et al. nevertheless claim that Mandarin-learning children produce higher proportions of verbs than do English-learning children; they estimate that the Mandarin learners produce approximately equal numbers of verbs and nouns.

Xuan and Dollaghan (2013) also examined English and Mandarin, but in their case with bilingual children (hence reducing child effects) using Communicative Development Inventories (CDIs). More verbs were produced by the same child in early Mandarin than early English. This study only included children who already had 50 spoken words, a relatively high level of spoken vocabulary for this type of study.

Childers et al. (2007) also noted no excess of nouns in first words using parent-completed inventories in Ngas, a Chadic language spoken in Northern Nigeria. They found comparably high ratios of verbs in comprehension; parent-completed inventories are ideal for comparing production and comprehension.

#### Linguistic Invariance in First Spoken Words

Some cross-linguistic data call these observed language specificities into question. The first group of studies quoted here have all used parent-completed inventories. Caselli et al. (1995) discuss the possibility that rich verb morphology, variable word order (including many verb-final child-directed utterances), and subject omission found in Italian might lead to earlier acquisition of verbs. Their data did not back this up: in both Italian and English, children used a preponderance of nouns in the first 50 words.

Looking only at the first 10 words produced, Tardif et al. (2008) suggest that names for people predominate in English, Mandarin, and Cantonese. Tardif et al. suggest that the classification of names for people as nouns is a mistake in this field. However, most other studies, cross-linguistic or otherwise, have concentrated on children with larger vocabularies.

Likewise, in Bornstein et al. (2004, an extensive cross-linguistic study of seven languages with differing sentence structures), a higher proportion of nouns than verbs was found in the vocabulary of 20 month old children, beyond the very first few words. The use of inventories may explain why this noun bias was found in all languages, even Korean. Other studies have also found no earlier verb production in Korean (Au et al., 1994; Kim et al., 2000) or Mandarin (Gentner, 1982).

Bornstein et al. (2004) suggest that child constraints (children’s pre-existing assumptions or knowledge), common to every child learning language, may lead to the pattern of early learned nouns and later learned verbs. Caselli et al. (1995) also conclude from their comparative study that children learning different languages all respond in a characteristic way to nouns and verbs in the ambient language.

Finally, using spontaneous speech data, Stoll et al. (2012) found that in Chintang, a highly agglutinative language in which verb arguments are optional (so verbs appear more frequently than nouns), early language learners were still seen to produce a higher proportion of nouns to verbs than were adults. Stoll et al. (2012) suggest that the complex verb system in Chintang leads to a relative reduction in the number of verbs produced by children. This is in contrast to the argument by other authors of the reverse (Caselli et al., 1995; Childers et al., 2007). Stoll et al. (2012) also note that most studies, even those that analyse spontaneous speech samples including adult speech, do not assess noun:verb ratios in input.

#### Cultural Variance in First Spoken Words

Children learning to speak the same language may not necessarily experience the same parenting or the same type of input. Bornstein and Cote (2005) examined vocabulary composition in 20-month-old children growing up in varied cultural locations: three languages (Spanish, Italian, and English) and two settings (urban and rural). Using the same methodology for all children and calculating nouns and verbs as a proportion of the words available for parents to select on the inventory, children aged 20 months studied by Bornstein and Cote (2005) in rural Italy produced equal proportions of nouns and verbs. This was in contrast to all other settings in this study, and also in contrast to the findings of the same researchers previously (Bornstein et al., 2004). Bornstein and Cote (2005) suggest that there are differences in rural versus urban parents’ use of verbs in child-directed speech – specifically more didactic use of verbs by rural parents (Camaioni et al., 1998).

In a more direct examination of cultural differences, Fernald and Morikawa (1993) found that American mothers’ more frequent object labeling led to their infants having more nouns in their vocabularies than Japanese infants, whose mothers used more social routines. Tamis-LeMonda et al. (2014) note that across several cultures maternal responsiveness has been seen to vary in ways that are known to affect infants’ acquisition of different types of language. Hence cultural factors influencing parenting affect both children’s acquisition, and mode of use, of early words.

#### Comprehension, Vocabulary Knowledge, and Pragmatic Constraints

I now turn to early vocabulary comprehension, which tends to have a greater proportion of verbs than does early vocabulary production (Bates, 1979). Caselli et al. (1995) suggest this may simply be an artifact of the types of experimental materials used to elicit comprehension behavior, which may include more verbs than the type of material used to elicit production.

Goldfield (2000), however, examined parents’ elicitations of children’s speech and actions in spontaneous speech samples. She found that there was a difference between action- and object-directed speech such that parents’ elicitations of comprehension were more likely to be designed to lead to their child performing an action than indicating an object, while elicitations of child speech were directed toward production of a noun rather than a verb. This difference between action- and object-directed speech may help to explain the bias in children’s early language production to nouns. This adds to the evidence that children’s early word production is not wholly representative of their early word knowledge. Parents’ utterance type seems to be very dependent on context: in book reading contexts, parents use more object-oriented utterances (Altınkamış et al., 2014), whereas more action-oriented utterances are used in toy play, and this seems to occur even in very verb-oriented or very noun-oriented languages.

#### Knowledge versus Comprehension

Many studies and reviews discuss ‘learning’ of first words without distinguishing between comprehension and production though most of the data on which these discussions rely are from studies of children’s first spoken words. Many of the learning mechanisms proposed imply underlying ‘knowledge’ or ‘learning’ of lexical concepts (Markman, 1990; Gleitman et al., 2005). These discussions heavily imply that comprehension develops in direct parallel.

Examining data on early comprehension from parent-completed inventories shows that the proportion of verbs in early comprehended words is higher than the proportion in early-produced words (Bates, 1979; Caselli et al., 1995; Childers et al., 2007). In addition to ensuring that the data in the current study – on two languages which have been little studied to date – are compatible with those from previous studies, the current study must ensure that the data from comprehension are directly comparable with those from production.

### Study Languages and Setting

Kiswahili and Kigiriama are two Eastern Bantu languages, both spoken in rural coastal Kenya. The languages are very closely related and have extremely similar grammatical structure; both languages have the noun classes found in Bantu languages (similar to grammatical gender), with verbs, adjectives, possessives and other parts of speech agreeing with the noun class of nouns. The two languages have very similar verb morphology: the same grammatical features are marked on the verb in each language, with similar or identical verb affixes. Rich inflections, and especially rich verb inflections, are found in these languages as in others (such as Italian, Caselli et al., 1995).

However, the two languages are largely not mutually intelligible, despite a large number of cognates (possibly over 80%, Alcock et al., 2015). Census and informal estimates of the number of native speakers are around 15 million for Kiswahili and 900,000 for Kigiriama (Simons and Fennig, 2017).

Like many other richly inflected languages, these languages have highly variable word order. The basic word order is SVO, any word order is grammatical though alternate word order is usually marked. Caselli et al. (1995) hypothesize that word order variation in the input may affect the timing of verbs acquisition in child vocabulary. Even where a language is in essence SVO, the verb is frequently in a salient sentence-initial or sentence final position.

Caselli et al. (1995) go on to hypothesize that subject omission may also lead to higher salience of verbs in infant-directed speech (IDS). Verbs will constitute a higher proportion of the input language for children. The two languages studied here both allow overt subject or object omission, increasing even further the proportion of utterances in IDS that consist only of a single verb.

Pragmatics may also influence children’s vocabulary learning (Goldfield, 2000) when utterances in IDS expect an action or speech in response. Social expectations of even children speaking their first words, in this society, like other rural areas of developing countries, may include a high degree of obedience. This could mean that children hear more spoken commands, designed to result in child actions.

Some relevant data are available from other languages spoken in similar settings. Though these are not from the languages in question, it is possible to hypothesize from other data whether children are likely to hear commands and/or other types of speech in their input, and potentially to gain some idea of relative proportions of different types of input utterances.

Stoll et al. (2012) observed some prompts to repeat an utterance directed by adults toward children, in Chintang (rural Nepal). The Kenyan spontaneous speech samples also have some examples of older children eliciting repetitions (Alcock et al., 2012, 2015), and Rabain-Jamin (1998) observed this type of routine with toddlers in West Africa.

In both Rabain–Jamin’s setting and another West African setting mothers differed from older children in the types of speech they directed to infants. While in both settings high proportions of imperatives or directives were used, mothers used more declaratives while older children used more imperatives. Both mothers and older children described, and asserted, while older children used more Wh-questions (Nwokah, 1987; Rabain-Jamin, 1998). Rabain–Jamin also observed that mothers reported speech more often for younger toddlers (16–22 months) and prompted directly more with older toddlers (24–28 months).

Likewise in South Africa Kvalsvig et al. (1991) found that in Zulu- and Xhosa-speaking families, adults and older children used commands when speaking to pre-schoolers (age five), and pre-schoolers also used commands when talking to infants and toddlers. All interlocutors frequently used other types of speech acts, including informational and question acts. Roughly equal proportions of commands and information utterances were seen. Deen (2003) noted that around 30% of verbs in IDS Nairobi dialect Kiswahili were grammatical imperatives but did not quantify other utterance types.

Hence in similar cultures, commands – requests involving a verb and eliciting action – are heard in children’s input and could potentially encourage verb comprehension in first words. Many other types of utterances are also heard, including direct prompts for repetition.

### Predictions

Taking into account findings from a language with similarly complex and salient verbs (Caselli et al., 1995), and data from this setting and similar societies where commands are given at least as often as in European IDS (Goldfield, 2000), I hypothesize that children learning these two closely related Eastern Bantu languages will produce more nouns in their first spoken words than other categories. In contrast the noun bias predicted for production will be smaller or absent in the case of comprehension.

I hypothesize that this bias in production is due at least in part to factors, possibly input factors, that differentially affect spoken words – in other words the bias is not present in underlying early word knowledge itself. Although the study design does not allow direct assessment of mechanisms that could determine the source of any difference between production and comprehension, a smaller or non-existent noun bias in comprehension will necessarily imply the same in knowledge.

### Methodology

The method used needs to work in this setting and be comparable both across production and comprehension and with previous studies. Parent-completed inventories have been validated both for comprehension and production of vocabulary (Fenson et al., 1994). In particular, parents can use them to accurately report comprehension vocabulary (Mills et al., 1993, 1997; Schafer, 2005; Styles and Plunkett, 2009).

Using parent-completed inventories to collect cross-linguistic vocabulary data, Bornstein et al. (2004) examined production vocabulary only, while Caselli et al. (1995) looked at production and comprehension. In order to ensure cross-linguistic comparability, the current study will rely for the most part on a replication of the methods of Caselli et al. (1995; see below for more details), but adding analyses using the Bornstein et al. (2004) method of correcting for the number of opportunities that parents have to choose a word in any given category. Since there are more nouns than verbs in most parent-completed vocabulary inventories, the number of nouns a child knows may be artificially inflated if this correction is not carried out.

Both Caselli et al. (1995) and Bornstein et al. (2004) administered CDIs in written format. The Kenyan CDIs were necessarily administered in interview format.

## Materials and Methods

### Participants and Materials

A total of 208 families with children aged 8–20 months (mean 12.99 months, SD 2.91), resident in Kilifi District, Coast Province, Kenya, took part in the study. Families were recruited through a periodic census of villages and homesteads in the area. Of these families 63 were predominantly Kiswahili-speaking and 145 were predominantly Kigiriama-speaking. Speakers of the two languages are usually resident in different villages and follow different religions, so children are exposed primarily to their home language in their village and at social occasions. Where more than one of the languages, or another language, was spoken by adults to children, these children were excluded from the study. However, most adults in the study area speak at least a little of both languages plus some English, so some code-switching occurs in these primarily monolingual homes.

Families were interviewed verbally with the Kiswahili or Kigiriama version of the MacArthur-Bates Communicative Development Inventory – Words and Gestures (Fenson et al., 1994), constructed and validated for this community (Alcock et al., 2015). Assessment of both production and comprehension with the CDI were found to have external validity. Validation included comparison of parent report of comprehension with children’s communicative behavior (gesture and object name comprehension) in a session at children’s homes. Note in particular we found a relationship between parent report of comprehension of specific words on the CDI and children’s comprehension in a testing session of those particular items (significant at the one-tailed level with *N* = 17). We also validated vocabulary production in older toddlers against spontaneous speech production taken from home recordings, and against a picture vocabulary test. This gives confidence that the tool is valid for measurement both of comprehension and production.

An interview version of the CDI has also been validated against the Bayley Scales of Infant Development (Mental) in another, similar illiterate population (Hamadani et al., 2010). Data for the current study were collected as part of larger study investigating the effect of HIV exposure on infant development; the data presented here are from children who were not known to be exposed to HIV.

### Vocabulary Categories and Word Ranking

The number of words in each vocabulary category on the inventory is shown in **Table 1**. The inventory has a total of 292 vocabulary items. These were categorized using the method of Caselli et al. (1995). This method initially categorizes words into four broader categories, followed by seven narrower categories: Nominals (Common nouns, Proper nouns, and Sound effects), Routines, Predicates (Verbs and Adjectives), and Function words.

**Table 1:** Number of words in each lexical category on the vocabulary inventory.

	Sound effects	Animals	Vehicles	Toys	Foods	Clothes	Body parts	Small household objects	
Number of words	15	15	5	10	39	13	15	34	
	Furniture and rooms	Outdoor items	Places to go	People’s names	Games and routines	Verbs	Adjectives	Function words	
Number of words	11	18	10	14	12	56	15	10	
Broader categories	Category: Nominals	Sub-category: Common nouns (includes some items e.g., *shop* from Places to Go)	Sub-category: Sound effects	Sub-category: People	Category: Routines	Category: Predicates	Sub-category: Verbs	Sub-category: Adjectives	Category: Function words (includes remaining items e.g., *there* from Places to Go)
	194	165	15	14	12	71	56	15	15

For each language and for production and comprehension the most frequent 50 words produced and the most frequent 50 words comprehended (the first 50 words by rank) were noted. This replicates the methods of Caselli et al. (1995).

## Results

### Categorization of First Fifty Words

**Table 2** shows the categorisation of all words ranked under 50 in production and **Table 3** shows the same figures for comprehension, by language. Exactly 50 words were ranked between 1 and 50 in comprehension for both languages. However, because several words can be (and were) ranked equally, the number of words ranked under 50 for production is not the same in each language. This means that the number of words with this rank is greater than 50 (63 for Kigiriama and 57 for Kiswahili). The total numbers in each word category in production are hence shown in **Table 2** scaled down to 50. The vocabulary items ranked 1 through 50 in each language in comprehension, 1 through 46 in Kigiriama and 1 through 44 in Kiswahili in production, are shown in the Appendix, together with a translation equivalent.

**Table 2:** Highest ranked 50 words in each language, categorized by word class – Count of words for Production.

		Language		Scaled to 50 words	
Category – broader categories	Category – narrower categories	Kigiriama	Kiswahili	Kigiriama	Kiswahili
Function words		1	0	1	0
Nominals		54	49	43	43
	Common nouns	33	31	26	27
	People	11	10	9	9
	Sound effects	10	8	8	7
Predicates		2	3	2	3
	Adjectives	1	2	1	2
	Verbs	1	1	1	1
Routines		6	5	5	4

**Table 3:** Highest ranked 50 words in each language, categorized by word class – Count of words for Comprehension.

		Language	
Category – broader categories	Category – narrower categories	Kigiriama	Kiswahili
Function words		0	1
Nominals		35	32
	Common nouns	24	23
	People	5	4
	Sound effects	6	5
Predicates		14	14
	Adjectives	1	2
	Verbs	13	12
Routines		1	3

Chi-square analysis revealed no significant differences in the categorisation of first words between the two languages, either in comprehension or in production, and with either broader or narrower categories. In addition, *t*-tests showed no differences in the total number of words produced or comprehended by children learning the two different languages; for production vocabulary *t*(206) = 0.751 and for comprehension *t*(206) = 0.873. Given the extremely high rate of cognates and the very closely related nature of the two languages, further data shown are from both languages, combined. It can be seen from these tables that, as in English and Italian, the majority of the earliest 50 words produced by children are nominals.

### Quantitative Vocabulary Growth in Comprehension and Production with Age

Data from only small numbers of children over the age of 16 months (the target maximum age for typically developing children for the original Words and Gestures inventory) were available, so for age analyses such children are excluded from the dataset. Mean vocabulary size of older children was within the range for the younger children, so their data were included in analyses of vocabulary categories by vocabulary size.

**FIGURE 1:**
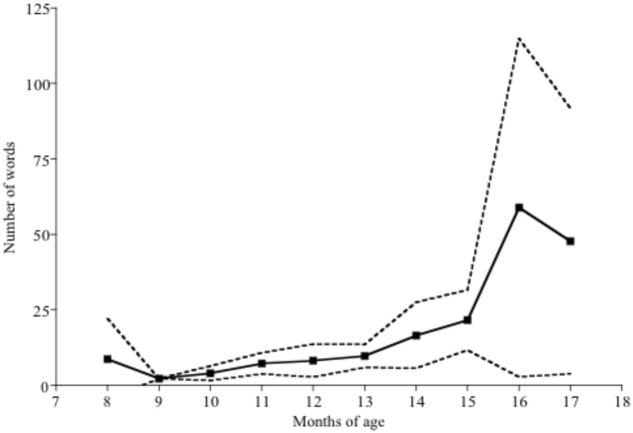
Number of words produced by children of each age, with 95% confidence intervals.

**FIGURE 2:**
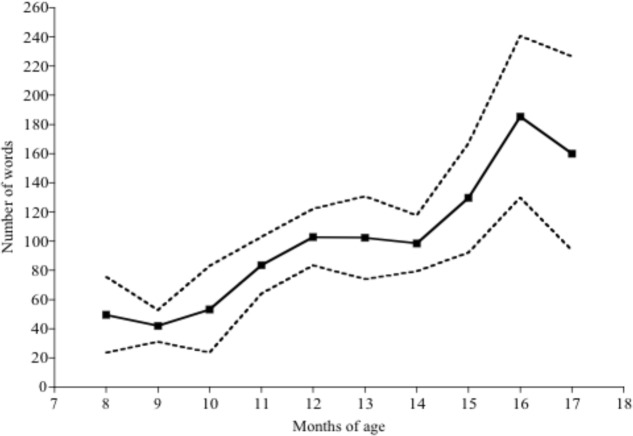
- Number of words comprehended by children of each age, with 95% confidence intervals.

The mean number of words produced and comprehended by children of each month of age can be seen in **Figures 1**, **2** respectively. Both production and comprehension vocabulary correlated significantly with children’s ages in months. For production vocabulary *r*(184) = 0.33, *p* < 0.001 and for comprehension vocabulary *r*(184) = 0.50, *p* < 0.001. Further details of the relationship between age and vocabulary are discussed in Alcock et al. (2015).

### Change in Categories as Vocabulary Grows

Analyses of the relationship between vocabulary categories and vocabulary size were planned and carried out as follows:

(1) Separate analyses of comprehension and production vocabularies:
(a) Simple percentage of all words known in comprehension and production in *broader* (Caselli et al., 1995’s Nominals, Predicates, Routines and Function words) and
(b) *narrower* (Common Nouns, Verbs, Adjectives and Function Words in Caselli et al. (1995) - Closed Class in Bornstein et al., 2004) word categories.
(c) Analyses computed as a proportion of the words in each category in the checklist – “chances to choose each category of word” – as in Bornstein et al. (2004) – *broader* categories of words, comprehension and production, for children with different vocabulary sizes and
(d) *narrower* categories of words, comprehension and production, for children with different vocabulary sizes.
(2) Combined analyses of comprehension and production vocabulary as in (c) and (d) only (proportion of chances – Bornstein et al., 2004).

### Analysis 1 – Nouns and Verbs in Early Production and Comprehension Vocabularies

Analysis 1a and 1b. Analysis of raw proportions of verbs and nouns in children’s comprehension and production vocabularies. Production: Here, nouns predominate in early production vocabulary with verbs forming a much smaller proportion of children’s early words – less than 10% in all vocabulary levels up to the 50 word production point. Data on both broader and narrower categories of production vocabulary can be seen in **Table 4**. As can be seen from this Table, the proportion of nominals in production starts at 96% in the smallest vocabulary group (1–5 words) and drops to 75% in the largest group (51+ words).

**Table 4:** Percentages of vocabulary consisting of words in each category, across vocabulary sizes – Production.

	Number of words in production vocabulary (N)					
	1–5 (82)	6–10 (35)	11–20 (41)	21–50 (16)	51+ (15)	Total
% Nominals	96.0	91.0	88.1	84.5	75.0	90.8
% Common nouns	2.7	13.6	23.6	43.4	59.1	17.0
% People	78.0	40.2	30.4	21.2	8.7	50.7
% Sound effects	15.5	37.1	34.2	20.0	7.2	23.2
% Routines	3.8	8.3	9.5	6.8	5.5	6.2
% Predicates	0.0	0.5	1.8	4.5	15.8	2.1
% Verbs	0.0	0.0	1.4	2.5	11.8	1.4
% Adjectives	0.0	0.5	0.4	2.0	4.0	0.7
% Function words	0.3	0.3	0.6	4.2	3.7	0.9

*Comprehension* can be seen in **Table 5**. Nominals are a smaller proportion of the vocabulary at all vocabulary sizes and verbs are over 15% of the vocabulary even at the smallest vocabulary size.

**Table 5:** Percentages of vocabulary consisting of words in each category, across vocabulary sizes – Comprehension.

	Number of words in comprehension vocabulary (N)						
	0–20 (20)	21–50 (43)	51–100 (62)	101–150 (40)	151–200 (26)	201+ (17)	Total
% Nominals	78.6	68.9	64.6	65.8	67.5	67.0	67.6
% Common nouns	38.7	46.0	48.3	53.9	57.6	58.2	50.0
% People	21.1	12.6	7.7	6.3	5.3	4.8	9.2
% Sound effects	18.8	10.6	8.6	5.6	4.6	4.1	8.5
% Routines	3.4	4.8	4.6	4.0	3.6	3.7	4.2
% Predicates	18.1	24.1	28.4	27.3	25.9	25.9	25.8
% Verbs	16.3	20.2	24.4	22.3	21.7	20.8	21.7
% Adjectives	1.8	3.9	4.0	5.0	4.4	5.1	4.1
% Function words	0.0	2.2	2.4	2.9	2.9	3.3	2.4

For the earliest stages (before the Kenyan children reach 50 words) the proportion of words that are verbs is very low in production. However after this stage (after 50 words) Eastern Bantu-learning children start to produce a mean of 11.8% of their words as verbs.

In comprehension, at a vocabulary level of under 21 words, the proportion of words that Eastern Bantu-learning children understood were 16.2% verbs. Nevertheless, in both production and comprehension the majority of words are nominals, at all vocabulary levels.

### Categorisation for Analyses 1c and 1d: Vocabulary Size Category Assignment

Bornstein et al. (2004) analyzed children’s production vocabulary by calculating vocabulary in each category of words as a proportion of the number of chances parents have to choose a word of that category – since in early vocabulary inventories, there are more nouns than other word types to choose from. The categories in the current study correspond to Bornstein et al.’s (2004) Nouns, Verbs, Adjectives and Closed Class.

Bornstein et al. (2004) analyzed data from somewhat older children (20 months) than in this paper, with larger vocabulary sizes. **Table 6** therefore shows vocabulary in each category as a proportion of available chances for children in the production vocabulary groups used in analyses 1a and 1b (ranging from 1 to 5 words to 51+ words). **Table 7** shows comprehension. Note these are not the same groups as in Bornstein et al. (2004) due to the smaller vocabulary size of the Kenyan children.

**Table 6:** Categories of words in production vocabulary at different vocabulary sizes expressed as a proportion of chances to choose each category.

Proportion of words on inventory	Number of words in production vocabulary (N)						
	1–5 (82)	6–10 (35)	11–20 (41)	21–50 (16)	1-50 (174)	51+ (15)	Total
Nominals	0.01	0.04	0.07	0.14	0.04	0.44	0.07
Common nouns	0.00	0.01	0.02	0.08	0.01	0.41	0.05
People	0.12	0.21	0.29	0.42	0.21	0.60	0.24
Sound effects	0.03	0.20	0.31	0.38	0.16	0.47	0.19
Routines	0.01	0.05	0.10	0.17	0.06	0.44	0.09
Predicates	0.00	0.00	0.00	0.02	0.00	0.25	0.02
Verbs	0.00	0.00	0.00	0.01	0.00	0.24	0.02
Adjectives	0.00	0.00	0.00	0.04	0.01	0.31	0.03
Function words	0.00	0.00	0.01	0.13	0.01	0.38	0.04

**Table 7:** Categories of words in comprehension vocabulary at different vocabulary sizes expressed as a proportion of chances to choose each category.

	Number of words in comprehension vocabulary (N)							
	0–20 (20)	21–50 (43)	51–100 (62)	101–150 (40)	151–200 (26)	1–200 (191)	201+ (17)	Total
Nominals	0.05	0.15	0.26	0.45	0.66	0.31	0.87	0.35
Common nouns	0.03	0.12	0.23	0.43	0.66	0.28	0.89	0.33
People	0.16	0.34	0.40	0.54	0.66	0.43	0.80	0.46
Sound effects	0.13	0.28	0.41	0.45	0.54	0.38	0.63	0.40
Routines	0.03	0.16	0.27	0.41	0.54	0.28	0.73	0.32
Predicates	0.04	0.13	0.29	0.47	0.65	0.32	0.85	0.36
Verbs	0.04	0.14	0.32	0.49	0.69	0.34	0.87	0.38
Adjectives	0.02	0.10	0.19	0.41	0.52	0.24	0.80	0.29
Function words	0.00	0.09	0.17	0.35	0.52	0.22	0.80	0.27

Children with 1–50 spoken words produced a mean of 4% of the nominals on the inventory compared with a mean of less than 1% of the verbs. Likewise children with larger vocabularies produced 44% of the nominals and only 24% of the verbs.

In comprehension, children with both smaller (<200 words) and larger (>200 words) comprehension vocabularies were reported to comprehend almost equal proportions of the nominals on the inventory (31% for the smaller vocabulary group and 87% for the larger vocabulary group) and verbs (34 and 87% respectively).

Analysis 1c. Analysis of broader categories of words produced and comprehended as a percentage of chances to choose those words, for children with different vocabulary sizes. ANOVAs were carried out to compare proportions of words on the inventory in each category produced versus comprehended by children in different vocabulary groups. These used the original four broad categories Nominals, Predicates, Routines and Function words.

For *production*, a significant main effect was found of word category, *F*(3,184) = 33.18, *p* < 0.001, η^2^ = 0.15. A significant interaction between word category and vocabulary group, *F*(12,184) = 5.00, *p* < 0.001, η^2^ = 0.10 was also found. For all ANOVAs post-hoc pairwise comparisons with Bonferroni corrections were carried out.

These pairwise comparisons showed that although the proportion of nominals did not differ significantly from that of routines, all other pairs were significantly different: parents reported a significantly higher proportion of nominals than predicates or function words, of routines than of predicates and function words, and of function words than of predicates, were produced. The differences between word categories became smaller as vocabularies became bigger, however.

For *comprehension*, a significant main effect of word category was again seen, *F*(3,202) = 9.85, *p* < 0.001, η^2^ = 0.05, but no significant interaction between vocabulary size and word category. As with the raw data analysis above, for comprehension there are no differences in vocabulary composition as comprehension vocabulary increases. Here pairwise comparisons showed significantly higher proportions of nominals than routines or function words, and of predicates than routines or function words, were comprehended, but there was no significant difference between the proportion of nominals and of predicates that were comprehended, nor between routines and function words.

Analysis 1d. Analysis of narrower categories of words produced and comprehended as a percentage of chances to choose those words, for children with different vocabulary sizes. Analyzed in this way, with narrower groups of words comparable to the analyses of Bornstein et al. (2004) the data also show a predominance of nouns in first words, especially in production. The sample in the current dataset is biased toward children with smaller vocabularies, so the proportions are not completely comparable to those of Bornstein et al. (2004) Nevertheless, taking the children with 51 or more words as the median group in the Bornstein et al. (2004) ‘smaller vocabularies’ group, the figure of slightly less than twice as many nouns (compared to noun-opportunities) versus verbs (compared to verb-opportunities), is similar to the figures for most of the languages in the Bornstein et al. (2004) data.

The picture for comprehension is different, however – children with smaller comprehension vocabularies – 1–200 words – comprehended 28% of the possible common nouns and a higher proportion, 34%, of the possible verbs. Children with comprehension vocabularies over 200 comprehended 89% of the possible common nouns and 87% of the possible verbs, though in this group a ceiling effect may be operating.

ANOVAs were carried out to examine growth of vocabulary in these categories as overall vocabulary sizes grow. For production, a significant effect of category, *F*(3,183) = 14.53, *p* < 0.001, η^2^ = 0.07 and an interaction between category and vocabulary level, *F*(12,183) = 4.31, *p* < 0.001, η^2^ = 0.09 were found. As children’s vocabularies grew, the proportions of different word classes produced became more similar.

**FIGURE 3:**
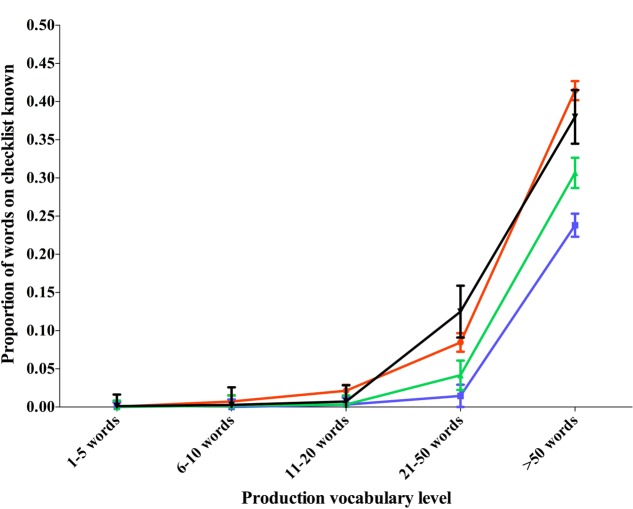
Proportion of words in different categories produced by children of different vocabulary levels.

**FIGURE 4:**
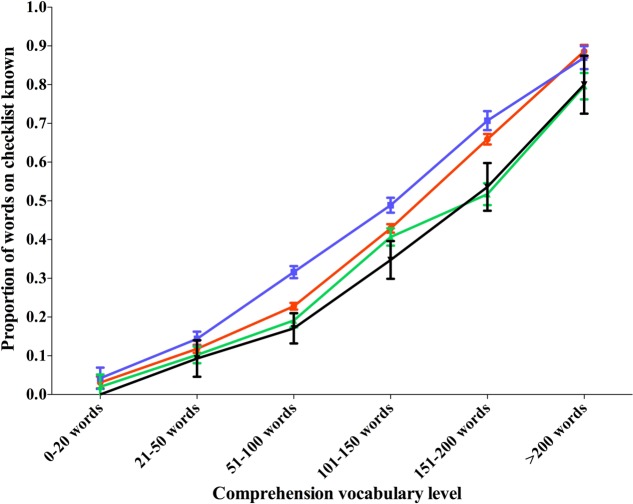
Proportion of words in different categories comprehended by children of different vocabulary levels.

For comprehension, again a significant effect of word category, *F*(3,201) = 12.60, *p* < 0.001, η^2^ = 0.06 was found, but as above there was no interaction; there is no change in the proportions of words in different categories as vocabulary grows. Data from these comparisons for production and comprehension can be seen in **Figures 3**, **4** respectively.

### Analysis 2 – Combined analysis of Comprehension and Production

Grand ANOVAs (combining previous analyses) were carried out to compare the proportions of the words on the checklist that can be seen in children’s production versus comprehension at different vocabulary sizes. As only one measure of vocabulary size can be used for this analysis, comprehension vocabulary size was chosen – all children had a comprehension vocabulary of 1 or more, while many had a production vocabulary of zero, reducing the variance. This means that these analyses are not precisely comparable to the separate analyses above.

Analysis 2c. Broader categories - Nominals, Predicates, Routines and Function words – Comparison of production and comprehension. Significant main effects of modality, word class, and vocabulary group were found, as well as significant interactions between modality and both vocabulary group and word class, and a three-way interaction between modality, vocabulary group and word class. Results of these two grand ANOVAs are shown in **Table 8**.

**Table 8:** Analyses of variance examining proportion of words known in different word classes in both comprehension and production modalities, as a function of vocabulary size.

Source	d.f	F	η^2^	P
	**Between subjects**			
Vocabulary group	5	129.53	0.76	<0.001
		150.48	0.79	<0.001
	**Within subjects**			
Modality	1	983.24	0.83	<0.001
		1104.91	0.85	<0.001
Word class	3	8.16	0.04	<0.001
				
Vocabulary group by modality	5	69.74	0.63	<0.001
		90.73	0.69	<0.001
Vocabulary group by word class	15	Not significant		
Modality by word class	3	18.68	0.08	<00.001
		20.21	0.09	<00.001
Vocabulary group by modality by word class	15	2.61	0.06	0.001
		Not significant		

*Cells show ANOVA 1 (Common Nouns, Routines, Predicates, Function Words) in the upper half and ANOVA 2 (Nominals, Verbs, Adjectives and Function Words) in the lower half. Degrees of freedom are the same for both ANOVAs*.

Planned comparisons showed differences between Nominals and Predicates (mean difference = 0.03, *SE* = 0.005, *p* < 0.001), and between Nominals and Function Words (mean difference = 0.06, *SE* = 0.016, *p* = 0.002).

Analysis 2d. Narrower categories - Common Nouns, Verbs, Adjectives, and Function words – Comparison of production and comprehension. Main effects were found of modality (comprehension versus production), word class and vocabulary group in addition to interactions between modality and both vocabulary group and word class. Planned comparisons showed significant differences between Common Nouns and Adjectives (mean difference = 0.04, SE = 0.007, p < 0.001) and between Verbs and Adjectives (mean difference = 0.04, SE = 0.007, p < 0.001).

Hence when comprehension and production are compared directly, the above findings are confirmed. As children’s vocabulary gets bigger, the proportion of words that they produce in different classes changes, but the proportion of words that they comprehend in different classes does not.

## Discussion

### The First Words that Children Say

When comparable techniques are used to investigate children whose input language varies, the first words that children say are predominantly nouns. This has been found in children who hear a variety of European, Asian and now African languages. The two extremely closely related Eastern Bantu languages studied here both allow sentences that consist of a single, highly inflected verb, as do Spanish or Italian. Such single-verb sentences may be even more common in Bantu than in Romance languages, since in Bantu languages subjects and objects can be represented as verb affixes. However, even single-verb sentences and highly variable word order do not lead children to produce verbs in large proportions in their first spoken words. Likewise, documented elicitations of other types of words from infants by older children might have led to lower proportions of nouns in first spoken words, but this is not the case.

This predominance of nouns in first spoken words holds up for children with vocabularies from 1 to 5 words up to more than 50 words. Early vocabulary checklists tend to contain a large predominance of nominals but nouns also predominate when the number of words in each category was analyzed as a proportion of chances to choose those categories of words in those categories. The results are the same, however, the words are categorized, too, whether as nouns versus verbs, adjectives and function words or whether of nominals versus predicates/function words.

As children’s spoken vocabularies grow, the proportion of words in different categories do change, however: there is a significant interaction between spoken vocabulary size and the proportion of words in each vocabulary categories. It is necessary to be cautious, though, in definitely categorizing children’s first spoken words as verbs or nouns. Even in languages where the surface forms of these are different, children may use a surface noun to represent an action, or a surface verb to represent an object associated with an action.

### The First Words that Children Understand

The picture is very different in comprehension, however. In the earliest words comprehended (1–20 words) nominals are also very common, but a higher percentage of words comprehended than words produced are verbs. At larger comprehension vocabularies, the proportion of words comprehended that are verbs increases slightly. Likewise, when analyzing percentage of chances to choose words in different categories, children at these levels of comprehension understand almost exactly the same percentage of the nouns and verbs on the checklist. As comprehension increases there is no significant change in the proportions of different types of words: the relative proportions of words in different classes remains the same as vocabulary grows.

### Directly Comparable Studies from Other Languages

Although the differences seen here between nouns and verbs and between production and comprehension are very similar to the differences found by Caselli et al. (1995) in both US English and Italian, production data from these Bantu languages may be more similar to the data from Italian than to that from US English. For example, among the first 50 words spoken in Italian are 8 words for people, compared with 9 in Kigiriama/Kiswahili and just 4 in US English. As suggested by Caselli et al. (1995), it is reasonable to conclude that this reflects the frequent contact which children in some societies have with extended family members.

There is a hint that verbs may be growing faster in early Kenyan children’s production vocabularies than in either US or Italian children’s production vocabularies. Children whose spoken vocabularies are greater than 50 words say fewer verbs in either US English or Italian than children learning Kiswahili or Kigiriama. When the number of words in each category was taken into account, Kenyan children in this spoken vocabulary group produced 41% of the common nouns on checklists and 24% of the verbs (a ratio of approximately 1.7:1). Looking at the Bornstein et al. (2004) data from older infants, for those with spoken vocabularies in the 51–100 word range, the ratio of noun:verb as a proportion of chances to choose words is very similar.

Between-language comparisons of the proportion of children’s vocabulary that is in each category are shown in **Table 9**. As discussed above, the proportion of nouns to verbs in early comprehension vocabulary does not seem to change as children increase their vocabularies in the Kenyan languages.

**Table 9:** Cross-linguistic comparisons of noun and verb use by children in the smallest and largest vocabulary groups.

Language	Comprehension – percentage of child’s vocabulary. Child comprehension 20 words or less		Production – percentage of child’s vocabulary. Child production 1–5 words	
	Nouns	Verbs	Nouns	Verbs
*Smallest vocabulary groups*
Kiswahili/Kigiriama	78.6	16.2	96.0	0.0
Italian	66.8	6.9	80.4	1.3
English	60.4	6.8	91.0	0.5

**Language**	**Comprehension – % of child’s vocabulary. Child comprehension more than 200 words**		**Production – % of child’s vocabulary. Child production more than 50 words**	
	**Nouns**	**Verbs**	**Nouns**	**Verbs**

*Largest vocabulary groups*				
Kiswahili/Kigiriama	67.0	20.8	75.0	11.8
Italian	60.7	17.5	72.6	6.8
English	61.8	16.0	73.6	4.5

*Data are from (Caselli et al., 1995)*.

Caselli et al. (1995) suggest that the excess of nouns over verbs in the construction of CDIs represents both an accurate reflection of the composition of adult vocabulary and of children’s early vocabulary – that children indeed first learn more nouns than verbs. Here this finding was replicated but only for production – not for comprehension.

More data on the actual proportion of nouns and verbs in the input language are needed. Stoll et al. (2012) examine this but few other articles attempt this comparison. But given the similar proportions found on checklists in many different, unrelated languages, and the preponderance of nouns in early production, it seems likely that the composition of many checklists genuinely corresponds to the composition of early spoken vocabulary. This does not appear to have been a strategy in checklist composition but rather a product of the exhaustive methods generally used to construct the checklists (Dale and Penfold, 2011). Indeed, it might be problematic if those constructing checklists decided *a priori* that they must contain differing proportions of words in different word classes. Researchers should still not forget that the composition of early comprehension vocabulary is not the same as the composition of early production vocabulary.

### Contrasting Findings from Other Languages

#### Production

There are a few studies that do not concur with these results. These include studies on Ngas, spoken in Nigeria, and on Mandarin.

Childers et al. (2007) suggested that the cultural context of child-rearing in Nigeria does not emphasize elicited labeling or object-directed behavior. Here children’s first words contained equal numbers of nouns and verbs. In rural Kenya, where caregivers are similarly often engaged in other activities and rarely participate in direct ostensive behavior with objects, older children are observed to attempt elicitations of all classes of words, and infants nevertheless still produced mainly nouns among their first spoken words.

Childers et al. (2007) suggest that children’s verb learning may also be enhanced in Ngas due to features such as single syllable words and regular, rich verb inflection (carried on separate function words). Italian, Spanish and these Eastern Bantu languages have this rich verb inflection (Caselli et al., 1995; Bornstein et al., 2004) but still nouns predominate in early spoken words.

The combination of cultural and grammatical features in Ngas may together drive early production of verbs; though it is difficult to see why the same factors do not produce the same results in the Kenyan languages. One point to note is that the Childers et al. (2007) CDI had a smaller number of words than in most other inventories, and has no sound effects. Sound effects are a major category of children’s early words, frequently used by both children and adults as spoken labels for objects (possibly due to auditory salience; Laing et al., 2016); in US English, Italian, and the Kenyan languages, children’s first spoken words contain 20–30% sound effects.

Childers et al. (2007) also suggest that relevant verb features may be operating in Mandarin (Tardif et al., 1999). The Mandarin data though suffer from a scaling problem – the children learning Mandarin had relatively large spoken vocabularies, double that of the children in the same study learning English, and though the study scaled children’s vocabulary, this leaves the composition of their vocabulary in doubt. Data from English and Dutch (Bornstein et al., 2004) do not demonstrate that monosyllabic verbs necessarily lead to early verb learning.

#### Comprehension

Data from other languages concur with these findings that more verbs are comprehended early than are spoken. However, some researchers have doubted parents’ abilities to report children’s comprehension vocabularies accurately (Houston-Price et al., 2007), but other data suggest parents can report comprehension (Mills et al., 1993, 1997; Styles and Plunkett, 2009), including our data on individual words reported on this CDI (Alcock et al., 2015). The main issue with accuracy seems to be that parents find reporting overall vocabulary size easier than reporting the precise words children know, especially as vocabulary increases. Given consistency between studies and between languages, where methodology is constant, it is likely that parents are also relatively accurate in reporting the classes of word that children comprehend.

One argument for using parental report for comprehension at lower levels of vocabulary only is that parents may become confused once children’s production vocabularies are larger. As children are less likely to produce verbs than nouns at lower levels of comprehension, parents may be more accurate in reporting the verbs. The structure of CDIs may also aid parents’ recall of comprehension in low-production categories such as verbs, since words of one class are generally all clustered together on CDIs.

Pragmatic processes also explain why children comprehend more verbs than they produce. Goldfield (2000) suggests that caregiver structuring of interactions gives children opportunities to demonstrate and practice production of nouns but comprehension of verbs. Children in other sub-Saharan African cultures hear a reasonable proportion of commands (i.e., verb comprehension opportunities) in IDS, but also hear a wide range of other types of utterances (Nwokah, 1987; Kvalsvig et al., 1991; Rabain-Jamin, 1998; Deen, 2003). If Goldfield’s explanation is valid, it implies that vocabulary knowledge may not differ between comprehension and production.

### Vocabulary Size

It is also helpful to consider whether children in this setting have comparable vocabulary levels to other settings, since verb/noun ratios depend on vocabulary size. In both production and comprehension mean vocabulary levels are intermediate between those found in UK English and those found in US English (Fenson et al., 1994; Hamilton et al., 2000). This is despite the extreme levels of poverty found in rural Kenya and the widely documented influence of poverty on early language and excess of children with language delay in low-income groups (see, for example Campbell et al., 2003).

## Summary and Conclusion

These data show that children hearing these two East African Bantu languages start by producing far more nouns than verbs but increase the proportion of verbs as their vocabulary increases. In contrast there is a more even distribution – and no real change with age – between these two important word classes in comprehension. Kenyan children show some signs of learning verbs earlier than children learning to speak other languages, but there is no indication that verbs predominate in these children’s first words as has been suggested for other languages (Brown, 1998; Tardif et al., 1999; Childers et al., 2007).

These findings imply that there may be no higher proportion of noun knowledge in early vocabulary, but simply a higher proportion of noun production. Explanations from pragmatics lend weight to this possibility. This has important implications for models of early word learning, including the ideas that nouns and/or object names are easier for children to learn. The factors that are hypothesized to assist in noun learning may still make nouns easier for children to produce, however.

The design of this study means that the data are comparable to those of Caselli et al. (1995) and to some extent to those of Bornstein et al. (2004). It is not possible to be as confident that the first words recorded here are genuinely comparable to those recorded by parents in the Tardif et al. (1999) study, where children’s vocabularies were much larger. Likewise the composition of the vocabulary checklist in the Childers et al. (2007) study is not directly comparable to this or other previous studies.

An interesting related point is the relationship between age, vocabulary size, and vocabulary composition. The Mandarin- and English-learning children in the Tardif et al. (1999) study were of the same age but different vocabulary sizes. In Bornstein et al.’s (2004) cross-linguistic study vocabulary was recorded for all of the children at the same age, while in this study and Caselli et al.’s (1995) study children were younger and of a variety of ages, but some of the children had comparable vocabulary sizes to those in Bornstein’s study. However, there are some indications that children with the same vocabulary size, speaking the same language, but of different ages, may have different vocabulary compositions (Rowland et al., 2016).

While studying this phenomenon in these languages is interesting in that little is known about vocabulary development in Eastern Bantu languages nor in children growing up in sub-Saharan African cultures, our study is not just of interest for this reason. Using an internationally accepted method of studying early language comprehension and production, but in understudied languages and a non-WEIRD (Henrich et al., 2010) setting, makes our findings – confirming and extending previous studies – additionally valid and, it can be argued, more interesting.

Many previous studies examining noun and verb learning in early language have not collected data on comprehension. The comparison here with English and Italian represents one of the few published studies of directly comparable data, with enough detail within the published article, to enable a direct comparison. A future larger-scale study such as that of Bornstein et al. (2004), but concentrating on younger children and either collecting additional data on comprehension, or utilizing one of the publicly available CDI datasets (Frank et al., 2017), could therefore be highly informative. The composition of vocabulary scales must though be directly comparable (avoiding issues such as the elimination of large, important early categories of vocabulary as in Childers et al., 2007), and the composition of the actual input language to children’s should also be a priority (Stoll et al., 2012).

## Ethics Statement

The Kenya Medical Research Institute National Scientific and Ethical Committees approved the study (SCC No: 832). Informed consent was obtained from all families and guardians of study participants. Because of the nature of the sample, and the number of illiterate parents, consent was obtained orally from many participants.

## Author Contributions

KA designed and implemented the study, supervised data collection, carried out the analysis and wrote the manuscript.

## Conflict of Interest Statement

The author declares that the research was conducted in the absence of any commercial or financial relationships that could be construed as a potential conflict of interest.
