# Supplementary material for: Production Is Only Half the Story — First Words in Two East African Languages
Source: Front Psychol. 2017 Oct 30;8:1898. doi: 10.3389/fpsyg.2017.01898 (PMC5676187; doi:10.3389/fpsyg.2017.01898)
Supplement: Supplementary file 1 [file Data_Sheet_1.pdf]

Appendix

First 50 words comprehended in Kigiriama and Kiswahili

Rank Kigiriama	Kigiriama word	English equivalent for Kigiriama	Rank Kiswahili	Kiswahili word	English equivalent for Kiswahili
1	Baba	Father	1	Mama	Mother
2	Mama	Mother	2	Baba	Father
3	Muswa	Porridge	3	Maji	Water
4	Madzi	Water	3	Uji	Porridge
5	Kelesi	Sit	3	Jina la mtoto mwenyewe	Child's own name
6	Mee mee	Goat noise	6	Mee mee	Goat noise
7	Lola	Look for	6	Gari/Motokaa	Car
8	Lala	Sleep	8	Miau miau	Cat noise
9	Mbuzi	Goat	8	Kikombe	Cup
9	Gari	Car	10	Mbuzi	Goat
11	Wari	Thick porridge	10	Kijiko	Spoon
12	moo moo	Cow noise	12	Paka/Nyau	Cat
12	Hala	Take	13	Vruum Vruum	Car
14	Vruum Vruum	Car noise	13	Maziwa	Milk
14	Dzina ra ye mwana mwenye	Child's own name	13	Mtoto	Child
14	Anwa	Drink	13	Shika	Catch, hold

14	Arya	Eat	13	Cheka/ Tabasamu	Laugh/ Smile
18	Kijiko	Spoon	18	Taa	Lamp
19	Tamu tamu/Pamu pamu	Sweet/ yum yum	19	Tamu tamu	Sweet/yum yum
19	Kuku	Chicken	19	Shh	Keep quiet
19	Mupira	Ball	21	Titi/Nono/Nyo nyo	Dummy/suck
22	Zaziga	Play	21	Piga teke	Kick
23	Hawe/ Nyanya	Grandmother	23	Tazama/angalia	Look/look at
24	Ng’ombe	Cow	24	Kinyago/Doli	Doll
24	Kikombe	Cup	24	Mpira/Boli	Ball
24	Nyamala	Quiet	24	Nyamaza	Be quiet
27	Paka/Nyau	Cat	27	Mdudu	Insect
27	Pamu	Sweet	27	Bisikuti	Biscuit
27	Ima	Stand	27	Chai	Tea
30	Mtsanga	Sand	30	Peremende	Sweets
31	Ukaleto/Bye	Bye	30	Keti	Sit
31	Luma	Bite	32	Moo moo	Cow noise
33	Piga makofi	Clap	32	La/Hapana/ Sitaki	No
34	Muhoho/ Dede	Child	32	Lala	Sleep
34	Gwira	Catch, hold	35	Cheza	Play
36	Basikili	Bicycle	36	Dawa	Medicine

37	Kuro	Dog	36	Nje	Outside
38	Beseni	Basin	36	Angusha	Drop
39	Bisikuti	Biscuit	39	Ng’ombe	Cow
40	Chakurya	Food	39	Leso	Sarong with motto
41	Lumira	Ouch, hurts	41	Kaa	Stay
41	Chai	Tea	41	Moto	Hot
41	Kitsana/ Shanua	Comb	43	Kuku	Chicken
41	Tsuha	Throw	43	Basikili	Bicycle
45	Miau miau	Cat noise	43	Simama	Stand
45	Reha	Bring	46	Ndizi	Banana
47	Maziya	Milk	46	Pole	Sorry
47	Kitanda	Bed	46	Ona	See
49	Maembe	Mango	46	Beba	Carry
50	Nguo	Clothes	50	Soksi	Sock

All words ranked below 50 in order of first production in Kigiriama and Kiswahili

Rank KiG	Kigiriama word	English equivalent for Kigiriama	Rank KiSw	Kiswahili word	English equivalent for Kiswahili
1	Baba	Father	1	Mama	Mother
2	Mama	Mother	2	Baba	Father
3	Moo moo	Cow noise	3	Mee mee	Goat noise
4	Mee mee	Goat noise	4	Tamu tamu	Yum yum
5	Tamu tamu/pamu pamu	Yum yum	5	Moo moo	Cow noise
6	Muhoho/dede	Child	5	Tamu	Sweet
7	Vruum vruum	Car noise	7	Mbuzi	Goat
8	Pamu	Sweet/tasty	8	Miau miau	Cat noise
9	Hawe/nyanya	Grandmother	8	Paka/nyau	Cat
10	Miau miau	Cat noise	8	Nyanya/Bibi	Grandmother
11	Tsawe/babu	Grandfather	11	Ng'ombe	Cow
12	Paka/nyau	Cat	11	Mtoto	Child
13	Asante/mumvera	Thank you	11	Babu	Grandfather
14	Eeeh	Yes	11	Kwa kheri/Bye bye	Bye
15	Huu huu huu	Dog noise	15	Ahsante/Shukrani	Thank you
16	Muswa	Porridge	16	Ndugu	Sibling
16	Wari	Thick porridge	17	Vruum vruum	Car noise
18	Madzi	Water	17	Titi/Nono/Nyonyo	Dummy/nipple/suck

19	Kokoikoo	Cockerel noise	19	Maji	Water
20	Ukaleto/bye	Bye bye	19	Kitoto	Infant
21	Kuku	Chicken	19	La/Hapana/Sitaki	No
21	Nyama	Meat	22	Mdudu	Insect
23	Haah	No	23	Huo huo	Dog noise
24	Mududu	Insect	23	Umia	Ouch/hurts
24	Hombo/nyonyo/kopo ra mwana	Nipple/dummy/suck	23	Chai	Tea
26	Ng'ombe	Cow	23	Mjomba/Uncle	Maternal uncle
26	Dzina ra kuro/paka	Dog or cat's name	27	Kuku	Chicken
26	Mwana mutsanga	Infant	27	Moto	Hot
29	Lumira	Ouch/hurts	29	Mpira	Ball
29	Gari	Car	29	Bisikuti	Biscuit
29	Mupira	Ball	29	Maziwa	Milk
29	Chai	Tea	29	Uji	Porridge
29	Maziya	Milk	29	Mambo?	How are you?
34	Baa baa	Sheep noise	34	Simu	Phone noise
34	Mbuzi	Goat	34	Gari	Car
34	Ahu/jomba/uncle	Maternal uncle	34	Ndizi	Banana
34	Dzina ra murezi	Caregiver's name	34	Nyama	Meat
38	Kunguru	Crow noise	34	Kijiko	Spoon
38	Izu	Banana	34	Peni/shilingi	Shilling /penny
38	Supu/mutsuzi	Soup	34	Taa	Lamp
38	Nguo	Cloth	34	Jina la mlezi	Caregiver's name
38	Redio	Radio	34	Jina la motto	Child's own name

				mwenyewe	
38	Dzina ra ye mwana mwenye	Child's own name	34	Chafu	Dirty
38	Mutu	Person	44	Mbwa	Dog
38	Busu/shumu	Kiss	44	Basikili	Bicycle
46	Kuro	Dog	44	Barafu	Ice pop
46	Doli	Doll	44	Kiazi	Potato
46	Bisikuti	Biscuit	44	Maembe	Mango
46	Kumbu	Sardine	44	Mkate	Bread
46	Maembe	Mango	44	Sima	Thick porridge
46	Mukahe	Bread	44	Suruali	Trousers
46	Yai/iji	Egg	44	Kikombe	Cup
46	Kijiko	Spoon	44	Kisu	Knife
46	Kikombe	Cup	44	Uchafu	Waste
46	Taa	Lamp	44	Naam/Ndio/Ehe	Yes
46	Kigongo	Stick	44	Cheka/Tabasamu	Laugh/smile
46	Mtsanga	Sand			
46	Panga	Machete			
46	Mambo/mautu?	How are you?			
46	Shh	Be quiet			
46	Moho	Hot			
46	Ii	This thing			
